# Supplementary material for: The association between maternal intra-abdominal pressure and hypertension in pregnancy
Source: PLoS One. 2023 Oct 18;18(10):e0284230. doi: 10.1371/journal.pone.0284230 (PMC10584176; doi:10.1371/journal.pone.0284230)
Supplement: Attachment — Submitted filename: Response to Reviewer # 2 Comments .docx [file pone.0284230.s001.docx]

**Re: Response to Reviewer # 2 Comments - “The association between maternal intra-abdominal pressure and hypertension in pregnancy” - PONE-D-23-06569**

**1. In Pregnancy induced hypertension one of the main physiological change in maternal body is fluid retention. Therefore I think it is important to find out association between maternal weight gain with Intra abdominal pressure. There could be increase abdominal pressure due to abdominal wall swelling! Also according to study result there is no correlation with fetal weight and abdominal hypertension in Pregnancy induced hypertension group.**

Agreed with your valuable comment. We conducted a cross sectional study. However, prospective cohort study design with serial measurement of IAP in different gestations and postpartum, maternal weight gain, fetal weight gain in HIP and normotensive groups may be more informative.

In our study, IAP is correlated with birth weight in normotensive pregnancy. Interestingly, It is not correlated with birthweight in HIP group. It raises the suspicious of “IAP in HIP group may not only result of growing fetus compare to the normotensive group – Additional increased IAP may play role in pathogenesis of HIP’’***.***

***This information is now included in the manuscript under Discussion - Line number 260-262***

**2. I think post partum pain management is very important in planning to measure abdominal pressure in both groups. Therefore it is very important to describe post partum pain management in study.**

As part of the post-CD analgesic protocol, effective pain management was ensured by consistently evaluating post-operative pain levels using the 0-10 visual analogue scale (VAS) and administering analgesics in accordance with the WHO analgesic ladder. IAP measurements were repeated between six to eight hours post-operatively. In cases where the VAS score was less than 3, IAP measurement was performed. However, if the VAS score was 3 or higher, analgesics were administered and IAP measurement was subsequently conducted after confirming a VAS score below 3.

***This information is now included in the manuscript under Methods - Line number 139-146***
